# PPAR-γ Partial Agonists in Disease-Fate Decision with Special Reference to Cancer

**DOI:** 10.3390/cells11203215

**Published:** 2022-10-13

**Authors:** Sangeeta Ballav, Bini Biswas, Vishal Kumar Sahu, Amit Ranjan, Soumya Basu

**Affiliations:** Cancer and Translational Research Centre, Dr. D. Y. Patil Biotechnology and Bioinformatics Institute, Dr. D. Y. Patil Vidyapeeth, Tathawade, Pune 411033, India

**Keywords:** PPAR-γ, partial agonist, TZDs, disease, cancer, type 2 diabetes mellitus, autoimmune, dermatology, cardiovascular disorders

## Abstract

Peroxisome proliferator-activated receptor-γ (PPAR-γ) has emerged as one of the most extensively studied transcription factors since its discovery in 1990, highlighting its importance in the etiology and treatment of numerous diseases involving various types of cancer, type 2 diabetes mellitus, autoimmune, dermatological and cardiovascular disorders. Ligands are regarded as the key determinant for the tissue-specific activation of PPAR-γ. However, the mechanism governing this process is merely a contradictory debate which is yet to be systematically researched. Either these receptors get weakly activated by endogenous or natural ligands or leads to a direct over-activation process by synthetic ligands, serving as complete full agonists. Therefore, fine-tuning on the action of PPAR-γ and more subtle modulation can be a rewarding approach which might open new avenues for the treatment of several diseases. In the recent era, researchers have sought to develop safer partial PPAR-γ agonists in order to dodge the toxicity induced by full agonists, akin to a balanced activation. With a particular reference to cancer, this review concentrates on the therapeutic role of partial agonists, especially in cancer treatment. Additionally, a timely examination of their efficacy on various other disease-fate decisions has been also discussed.

## 1. Introduction

**Figure 1:**
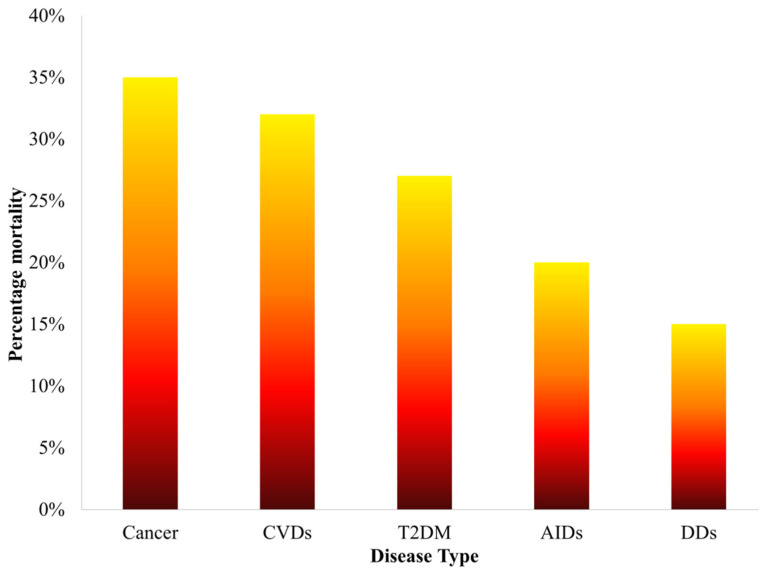
Statistics of percentage mortality caused by various diseases over the past five years.

Disease complexities involving cancer, cardiovascular diseases (CVDs), type 2 diabetes mellitus (T2DM), autoimmune disorders (AIDs), namely, multiple sclerosis, rheumatoid arthritis and autoimmune thyroid diseases and dermatological diseases (DDs) are significantly linked to morbidity and mortality around the world. Although the survival rates for these diseases are rising, they have a noteworthy mortality rate of 25% worldwide, wherein cancer stands at 35% [1], CVDs at 32% [2], T2DM at 27% [3], AIDs at 20% [4], and DDs at 15% [5] (Figure 1). Thus, the statistics depict the severeness of these illnesses in a susceptible cohort of individuals over the last 5 years. The aforementioned diseases may coexist and share significant risk factors as well as pathophysiological mechanisms. This has opened doors to decipher new therapeutic approaches common to all the outlined disorders. Peroxisome proliferator-activated receptor (PPAR) has been an underlying transcription factor highlighting its importance in numerous diseases including cancer, CVDs, T2DM, AIDs and DDs [6,7,8]. Being a member of the nuclear hormone receptor (NHR) superfamily, PPAR is primarily known to serve as a regulator of glucose metabolism and homeostasis [9].

The NHR superfamily comprises a group of transcription factors (TFs) that participate in a variety of critical biological mechanisms such as cellular respiration, reproduction and inflammatory processes. The foremost member of this superfamily was cloned in 1985; however, it now contains around 48 members in mammals, specifically humans [10]. The majority of NHRs are controlled by naturally occurring lipophilic ligands such as retinoids, phospholipids, and steroids. The ability of PPAR to cause hepatic peroxisome proliferation in mice in response to xenobiotic stimuli gave rise to the family’s name [11]. Research on the structure and function of the three human PPAR isotypes, namely PPAR-alpha (PPAR-α), PPAR-beta/delta (PPAR-β/δ), and PPAR-gamma (PPAR-γ), have confirmed their crucial roles in regulating carbohydrate and lipid metabolism as well as nutrition sensing. While PPAR-α and PPAR-β/δ appear to be primarily driving oxidative lipid metabolism, PPAR-γ is predominantly engaged in the cellular digestion of lipids via anabolic processes (Table 1), which also demonstrates its functional diversity in regulating glucose metabolism, homeostasis, adipocyte differentiation and cellular maintenance [9].

PPAR-γ performs the process of transcription in the nucleus after getting bound with ligand, forming a heterodimeric complex with another NHR, retinoid-X-receptor (RXR). They are locally present in cytoplasm and translocate in nucleus after the stimulation of transcription. Later, this transcriptional complex, PPAR-γ-RXR binds to the enhancer region of PPAR response elements (PPRE) that initiates the transcription of target genes [12]. Therefore, ligands are regarded as the key determinant for tissue-specific activation of PPAR-γ protein receptor. These ligands can be categorized into endogenous, natural or synthetic. However, the mechanism that governs this process is merely a contradictory debate which is yet to be systematically researched. Either these receptors get weakly activated by endogenous or natural ligands [13] or over-activated by synthetic ligands which serve as complete full agonists [14]. Therefore, a moderate activation may serve to be a novel and intriguing therapeutic strategy which would encompass balanced PPAR-γ activity. Numerous partial agonists of PPAR-γ offer an exciting prospect for different diseases. To elucidate the therapeutic role of PPAR-γ modulators in aforementioned diseases, this review summarizes various agonists and endogenous, natural and synthetic ligands involved in the activation of the aforementioned protein receptor and their effect on different disease-fate decisions. With a particular reference to cancer, as the statistics highlights the highest mortality rate of the same, this review concentrates on the therapeutic role of various PPAR-γ agonists in the treatment of cancer. Additionally, with a special focus on partial agonists, a timely examination on their efficacy and how they can be far more effective than natural and synthetic agonists have been also discussed.

**Table 1:** Overview of the basic metabolic function of PPAR isotypes.

PPAR Isotype	Site of Elevated Expression	Cellular Mechanisms Initiated	Biological Function	Genes Targeted	References
PPAR-α	Heart, liver and kidney	β-oxidation of fatty acids, synthesis of lipoprotein and regulating metabolic pathways of amino acids	Complementation of metabolic reaction to fasting	Hydroxymethylglutaryl CoA synthase 2 (HMG-CoA S2)	[15]
PPAR-β/δ	Adipocytes and macrophages	Differentiation pathways of adipocytes and production of triglycerides	Differentiation pathways of adipocytes and fatty acid trapping	Fatty acid binding protein 4 (FABP4)	[16]
PPAR-γ	Adipocytes, skin and brain	β-oxidation of fatty acids	Coordination of muscle fibers and determination of its types	Acyl CoA Oxidase (AOX)	[17]

## 2. Structural Mechanism of PPAR-γ

**Figure 2:**
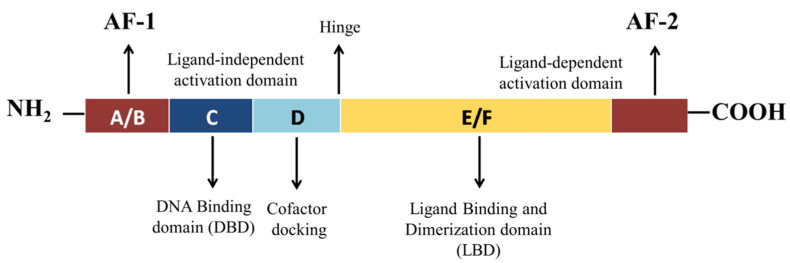
Schematic representation of functional domains of PPAR-γ. It comprises N-terminal AF-1 (ligand-independent activation domain) A/B domain, DNA-binding domain or the C domain) with two zinc fingers, a hinge (D domain), and C-terminal AF-2 (ligand-binding domain) E/F domain.

**Figure 3:**
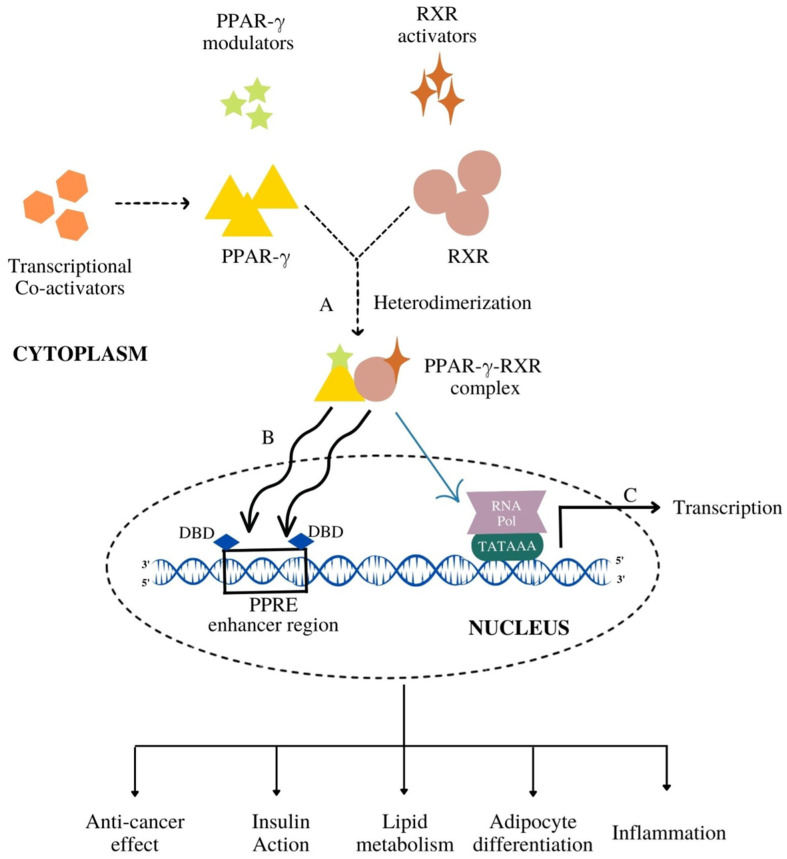
The mechanism of PPAR-γ activation followed by the gene transcription. (**A**) PPAR-γ upon binding with its modulators (endogenous, natural, synthetic) heterodimerize with RXR to form PPAR-γ-RXR heterodimerized complex. (**B**) This complex gets translocated inside the nucleus and binds to the DNA-binding domain (DBD) of PPAR response elements (PPRE) enhancer region. (**C**) Such a binding leads to the transcriptional activation of target genes resulting in various metabolic cascades such as insulin action, lipid metabolism and anti-cancer effects.

Like the other nuclear receptors, there are five functional domains present in the transcription apparatus of PPAR-γ: an amino terminal transactivation domain (activation function (AF)-1) with highly conserved DNA binding domain (DBD) that conducts the binding of heterodimerized complex to the promoter regions of the target gene, a hinge region and a carboxy terminus transactivation domain (AF-2) which encompass the ligand binding domain (LBD), which is 200–300 amino acids long, with a volume of 1440 Å^3^ (Figure 2). As the volume of LBD is large, it can bind and interact with a diverse group of ligands. The structural diversity of PPAR-γ sense varying ligands (weak or high affinity), and further, the receptor responds to environmental changes, such as metabolic changes or food intake. Along with this, it consists of 11–13 α-helices sandwiched in a three-layer anti-parallel helical structure and a four-stranded β-sheet that folds and becomes a large hydrophobic cavity which facilitate the binding of ligand to the receptor gene [18]. The transcriptional complex (PPAR-γ-RXR) induces a conformational change in transactivation domain at carboxyl terminus (AF-2) [19], followed by recruitment of certain co-activators with the release of co-repressors. After the shuttling of PPAR into nucleus, they associate with its various other transcription apparatus (Figure 3).

The conformational change induced by LBD triggers the accompanying process of co-activators (active conformations) and co-repressors (repressive conformations). Generally, a diverse group of co-activators, namely, p160/steroid receptor co-activator (SRC) family (SRC1–SRC3), cyclic-AMP (cAMP)-responsive element binding protein (CREB), CREB-binding protein (CBP) and its homologue p300, PPAR-binding protein (PBP) and PPAR-γ co-activator (PGC-1) have been adding vital impact in regulation of PPAR-γ transcriptional activation [20,21]. A typical co-activator comprises one or more LXXLL motifs (L: leucine and X: any amino acid) in the distal C-terminal region that facilitate the ligand-bound-PPAR to undergo its process. A growing body of evidence points to the organized assembly of these co-activators which is greatly influenced upon ligand binding to PPAR-γ [22]. A computational study determined the agonist mode of non-polymer ligand AZ 242 (Tesaglitazar) for PPAR-α and PPAR-γ which showed that AZ 242 activated both the receptors with the recruitment of SRC-1 co-activator. AZ 242 is a dihydrocinnamate derivative that is known to be an effective drug for abnormalities associated with glucose and lipid metabolism for insulin [23].

Along with co-activators, co-repressors also have been seen to modulate PPAR-γ transcriptional activity. Distinct co-repressors such as nuclear receptor co-repressor (NCoR) [24] and the silencing mediator of retinoid and thyroid hormone receptors (SMRT) [25] are destined to unliganded PPARs which repress the gene expression upon the recruitment of co-activators. Further, the structural transition leads to heterodimerization of the complex to achieve A PPAR-γ-RXR complex that binds strongly to PPRE [26]. This heterodimer complex recognizes the sequences located within six nucleotides of direct repeat spaced by one nucleotide (DR1) of PPRE which consists of a repeated sequence of AGGTCA (hexameric DNA consensus sequence) [27].

## 3. Functional Diversity of PPAR-γ

The nuclear receptor PPAR-γ gene on chromosome 3p25.2 in humans was identified as the master regulator of adipose tissue biology by Tontonoz and colleagues, in the early 1990s [28]. By using distinct promoters and alternative splicing mechanism, the human PPAR-γ gene possess nine exons that produce four PPAR-γ splice variants (PPAR-γ1-4), each code for one of two protein isoforms [29]. However, the mRNA translation of three splice variants, PPAR-γ1, γ3 and γ4, is regulated simultaneously and gets combined, forming one common isoform. Thusly, only two isoforms are considered, namely, PPAR-γ1 and γ2. PPAR-γ1 (477 amino acids) and PPAR-γ2 (505 amino acids) use two distinct promoters and alternate splicing for encoding the same gene. The absorbing difference between these two sub-isoforms lies in their amino-terminal A/B domain. The PPAR-γ1 isoform is produced by the mRNAs, PPAR-γ1, PPAR-γ3, and PPAR-γ4 [11]. PPAR-γ1 protein is widely expressed and found in skeletal muscle, liver, colon, cardiac tissue, adipose tissue, and many epithelial cell types. Additionally, PPAR-γ1 is expressed in numerous immune system cells, including T lymphocytes, dendritic cells, and monocytes and macrophages. The PPAR-γ2 isoform is produced via the translation of PPAR-γ2 mRNA transcript. Adipose tissue is the primary site of expression for PPAR-γ2, which has an extra 28 amino acids in its NH2-terminus. In addition to regulatory T cells (Tregs) and other T cell populations, PPAR-γ2 is also expressed in transitional epithelial cells of the urinary system’s organs, including the bladder [30]. Total PPAR-γ expression is, however, low in non-Tregs. PPAR-γ plays a crucial function at the nexus of immunity, obesity and cancer (Figure 3). In order to understand the shared and distinctive molecular mechanism underlying the functions of PPAR-γ, we need to assess its critical biological function in detail [31].

### 3.1. Anti-Cancer Effect

PPAR-γ is frequently expressed in various cancer cells, including breast, lung, colon, lips, kidney, pancreatic and thyroid [32]. Several studies have revealed that PPAR-γ activation by its agonists imposes cell cycle arrest [33], apoptosis [34], angiogenesis [35], inhibition [36], and redifferentiation [37], which are the key molecular processes associated with the prevention of tumor growth and progression. The expression of angiogenesis-related proteins such as vascular endothelial growth factor (VEGF) and cyclooxygenase-2 as well as inflammatory mediators in the tumor microenvironment are all inhibited by PPAR-γ activation [38]. The stimulation of cellular differentiation by PPAR-γ activation may serve as a tumor suppressor approach. In vivo experiments suggest that PPAR-γ interacts with downstream signaling pathways of the insulin-like growth factor (IGF) axis that involves mitogen-activated protein kinase (MAPK), phosphatidylinositol 3-kinase (PI3K), and the mammalian target of rapamycin (mTOR) cascades [39,40,41]. According to the anti-tumor function of PPAR-γ and the anti-apoptotic function of IGF system, MAPK activation by various growth stimuli such as insulin, IGF, epidermal growth factor (EGF), and platelet-derived growth factor (PDGF) may cause serine phosphorylation of PPAR-γ. This interference between IGF system and PPAR-γ phosphorylation constitutes various metabolic actions, such as lowered insulin level in blood and increased insulin sensitivity. As a result, this leads to the inactivation of its genomic activity [42].

PPAR-γ agonists may also encourage the differentiation of cancer cells. Experimental studies in cancer cells from the thyroid, breast, and lung cancers have demonstrated that thiazolidinedione (TZD) alters the epithelial expression profile and reverses the epithelial-mesenchymal transition (EMT) process [32,43]. Additionally, various studies have shown that PPAR-γ agonists are effective in preventing the cancer stem cells (CSCs) survival, obtained from human cell lines or samples of breast, prostate, colon, bladder, and venous tissues. This evidence supports the role of PPAR-γ agonists in controlling the biology of CSCs [44,45]. As previously indicated, the IGF pathway plays a critical role in the regulation of pluripotency, EMT, and self-renewal, which is crucial for the growth and expansion of CSCs. Gianì and collaborators have recently discovered that IGF-insulin receptor (IGF-IR), which controls the capacity for self-renewal and stem cell progression, is overexpressed in human thyroid progenitor/stem cells [46]. The IGF system is crucial for controlling stem cell biology and the early stages of the carcinogenesis process, as evidenced by similar findings in cancer progenitor/stem cells from solid and hematopoietic malignancies [47]. It should be noted that the molecular mechanisms by which TZDs control differentiation and stemness processes in adipocytes and normal cells have been investigated, although in cancer cells and CSC, they are still not fully understood [32].

### 3.2. Insulin Action

Adipose tissue combines cerebral and peripheral metabolic signals that control energy balance as a key tissue for whole-body energy homeostasis. They expand in response to an imbalance between energy intake and expenditure, which is characterized by an increase in cell size (hypertrophy) and cell number (hyperplasia) [48]. The underlying biological mechanism by which multipotent mesenchymal precursor cells commit to the adipocyte lineage and exhibit the usual hallmarks of mature fat cells is represented by a complicated and yet poorly understood set of transcriptional events. A number of distinct transcriptional factors have recently been found to control the expression of a group of genes involved in lipid and glucose metabolism. PPAR-γ, being one of them, have been demonstrated to play crucial role in the transcriptional regulation of genes encoding proteins involved in the aforementioned activities [9]. According to Leonardini et al. the treatment strategies of metabolic diseases linked to adipose tissue expansion, T2DM presupposes the identification and thorough comprehension of the molecular processes. They regulate these disorders, as well as the creation of therapies that specifically target the contributing elements [49].

Even in the absence of obese conditions, ageing and insulin resistance are linked to escalating flaws in mitochondrial oxidation. The elevated levels of fatty acetyl-CoA and diacylglycerol produced by this mitochondrial change can affect insulin signaling in skeletal muscle and other tissues, leading to insulin resistance [50]. PPAR-γ co-activators are thought to express less in people with insulin resistance. This results in fewer muscle mitochondria and a lower ratio of type 1 oxidative muscle fibers to type 2 fibers, which eventually becomes more glycolytic and possess fewer mitochondria [51]. Both non-diabetic and diabetic whites and Mexican Americans have been found to undergo these changes. PPAR-γ is considered as the molecular target of a series of insulin-sensitizing medications known as thiazolidinediones (TZDs) and is essential for maintaining glucose homeostasis [52]. The first synthetic PPAR-γ ligand was troglitazone; however, it was discontinued from usage due to rare but substantial liver damage. Rosiglitazone and pioglitazone are the two clinically available PPAR-γ ligands that are frequently used to treat T2DM [53]. TZDs lessen peripheral insulin resistance, which is a feature of T2DM patients. The overall glycemic control is improved as a result of the increased peripheral glucose utilization, decreased hepatic glucose output, and decreased blood glucose levels [54]. In addition, PPAR-γ ligands also lower plasma fat levels and improve glucose metabolism. Both pioglitazone and rosiglitazone raise high-density lipoprotein (HDL) levels in the serum, while pioglitazone significantly lowers triglyceride levels in the blood [55]. Additionally, PPAR-γ ligands prevent the production of numerous pro-inflammatory genes in macrophages, such as matrix metalloproteinases, interleukins, and inducible nitric oxide synthase. Since the gene expression and accumulation of macrophage in adipose tissue has been found to play in the pathophysiology of obesity-related insulin resistance, these actions may also be pertinent source for obesity-related insulin resistance [56].

### 3.3. Lipid Metabolism

Liver is a significant organ that regulates lipid intake from the circulatory system, de novo synthesis, and distribution of the generated lipids in the form of very low-density lipoprotein (VLDL) to peripheral tissues, thus controlling total body lipid homeostasis. White adipose tissue is thought to be the main organ for storing additional lipids, even though the liver regulates the body’s overall lipid balance [57]. In order to maintain and control homeostatic lipid transports under normal circumstances, the liver thus fulfills the aforementioned particular duties and is not meant to accumulate fat. However, unbalanced lipid flow in the liver causes fat buildup in pathophysiological diseases such as diabetes and obesity. Yoon Kwang Lee and collaborators confirmed in their study that it is the involvement of PPAR-γ in the hepatic fat buildup in pathologic circumstances, and hence, we discuss its critical role in lipid metabolism [58].

Overexpression of PPAR-γ1 or PPAR-γ2 strongly increases fat storage in the liver, which is expected to lead its adipogenic activity in white adipocytes [58]. Although the two PPAR-γ isoforms share the common DNA binding specificity, they exhibit different fat accumulation efficacy due to the variation in their transcription activity, wherein PPAR-γ2 has a five- to ten-fold higher transcription activity than PPAR-γ1. This is due to the additional amino acids at the N-terminus of PPAR-γ2 [59]. It has been established that PPAR-γ overexpression is brought by a high-fat diet (HFD) or other pathophysiological stresses that eventually causes lipid buildup in the liver. This is the first stage for the onset of non-alcoholic fatty liver disease (NAFLD), T2DM [60]. Given its direct involvement in the formation of lipid droplets, PPAR-γ might act as a defense against the lipotoxicity caused by free fatty acids (FFAs) and their derivatives. Under normal settings, hepatic PPAR-γ2 expression remains low, allowing the liver to transport freshly generated and/or dietary FAs in the form of VLDL to other peripheral tissues as an energy source. By storing the extra FFAs as triglycerides (TGs), hepatic PPAR-γ2 expression is up-regulated in response to HFD. This appears to protect the surrounding tissues from lipotoxicity and would be a typical procedure to counteract the continual flux of fat in the circulation [58].

### 3.4. Adipocyte Differentiation

Mammalian adipocytes have been identified as white, beige, and brown. Despite the fact that they all originate from separate progenitors and have fundamentally diverse morphologies and functions, the adipocytes undergo a well-coordinated differentiation process to develop into mature, fully functional cells. PPAR-γ has a well-established role in the longevity of adipocytes at various stages [31]. Non-hematopoietic pluripotent stem cells with a strong capacity for self-renewal and multi-differentiation activities are known as mesenchymal stem cells (MSCs). They develop into certain somatic cells of each hypoderm under normal differentiating process [61]. There are two distinct stages in adipocyte differentiation. Firstly, the MSCs begin to differentiate into precursor, adipocyte which is the crucial stage after receiving external stimulus. Molecular pathways underlying this particular mechanism are still unknown. Secondly, terminal differentiation involves three stages: proliferative, differentiative, and maturation phase, wherein the precursor adipocytes get transformed into mature adipocytes [61]. In the proliferative stage, the precursor adipocytes fuse and undergo contact inhibition followed by upregulation of genes, namely, CCAAT-enhancer-binding proteins (C/EBP β, C/EBP δ), Myogenic factor 5 (Myf5), before entering the cell cycle. Cell differentiation starts when the PPAR-γ binds to its respective ligands, e.g., TZD and its subtypes. Resting cells transition into the growth phase during the terminal differentiation stage, and the expression of related genes is activated by high levels of PPAR-γ. The cells finally develop into mature adipocytes after accumulating lipid droplets. The mature adipocyte stage is characterized by the presence of a single, big fat droplet inside the cell, strong expression of adipocyte marker genes such as aP2 and adiponectin, and production of cytokines that control energy balance, insulin sensitivity, and other activities [62].

### 3.5. Inflammation

**Figure 4:**
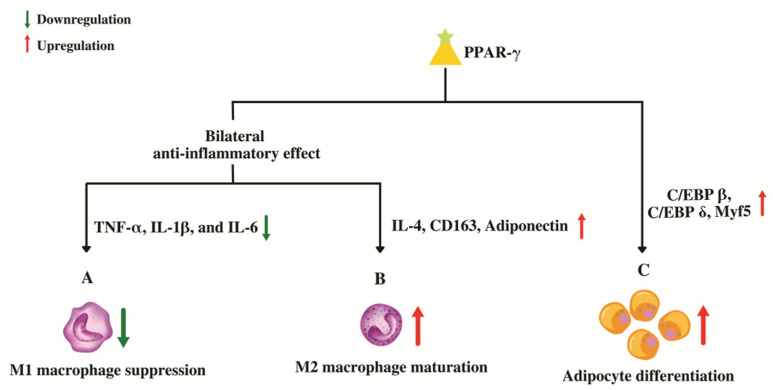
Role of PPAR-γ in macrophage conversion and adipocyte differentiation. (**A**) PPAR-γ suppresses the genes that code for pro-inflammatory molecules, which in-turn prevents the maturation of pro-inflammatory wild-type “M1” macrophages. (**B**) PPAR-γ promotes the maturation of anti-inflammatory “M2” macrophages by up-regulating anti-inflammatory genes leading to an overall bilateral anti-inflammatory effect. (**C**) PPAR-γ regulates adipocyte differentiation by upregulating the genes involved in proliferative stage of terminal differentiation.

As a member of the PPAR superfamily, PPAR-γ also controls inflammatory and immune reactions. PPAR-γ might stimulate trans-repression on pro-inflammatory genes genetically through SUMOylation or the conjugation of PPAR-γ with Small Ubiquitin-like Modifier (SUMO), a type of post-translational modification. Target genes for nuclear factor kappa B (NF-κB) are trans-repressed after the binding of a nuclear co-repressor complex, which keeps NF-κB in a repressed and promoter-bound state [63]. PPAR-γ may also control the immune system by virtue of dendritic cells (DC) and macrophages. Antigen acquisition, processing, stimulation, migration, cytokine generation, and lipid antigen presentation are all altered, which has an impact on the function of DC. While promoting the expression of anti-inflammatory mediators in macrophages, PPAR-γ suppresses the genes, namely, tumor necrosis factor alpha (TNF-α), Interleukin 1 beta (IL-1β), and Interleukin-6 (IL-6) that code for pro-inflammatory molecules [64]. Additionally, by altering cell differentiation, it prevents the maturation of pro-inflammatory wild-type “M1” macrophages by upregulating genes such as IL-4, CD163 and adiponectin that promote the development of anti-inflammatory “M2” macrophages, producing a bilateral anti-inflammatory effect (Figure 4) [64].

By enhancing tight junction proteins, PPAR-γ has been also shown to defend the blood–brain barrier’s (BBB) integrity. Through the measurement of NF-κB transcriptional activity and FITC-Dextran permeability, Talé and colleagues evaluated the impact of the PPAR-γ agonist, pioglitazone on markers of the inflammatory response and permeability changes brought on by hyperglycemia [64]. Their results indicated that high glucose-mediated activation of NF-κB is blocked by PPAR-γ agonist. Additionally, PPAR-γ agonists guard against FITC-Dextran permeability elevation that is aggravated by hyperglycemic conditions viz. T2DM. This shows that PPAR-γ has a protective function against the inflammatory and permeability changes that are caused by hyperglycemia in endothelial cells. It is noteworthy that mild PPAR-γ activators such as telmisartan, amorfrutin, and other PPAR-γ partial agonists significantly defend against PPAR-γ gene expression downregulation brought on by hyperglycemia [64].

## 4. PPAR-γ Activation by Various Ligands

**Figure 5:**
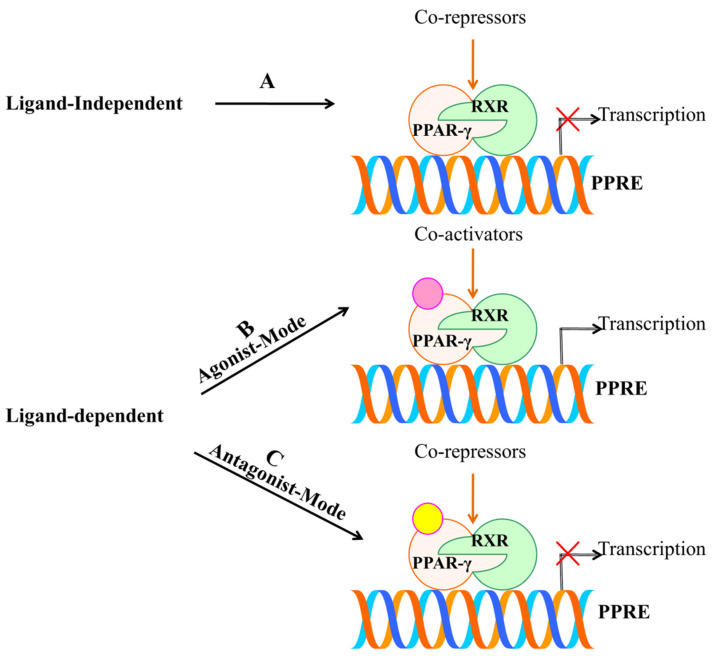
Schematic representation of activation of PPAR-γ receptor through ligand-dependent or -independent mechanisms. (**A**) Ligand-independent mechanism does not involve the active participation of ligand, and co-repressors bind to unliganded PPAR-γ, which repress the gene expression by chromatin remodeling. (**B**) Agonist mode of action involves the binding of ligand to LBD of PPAR-γ with the help of co-activators, which further leads to transcription of targeted genes. (**C**) Antagonist mode of action also involves the binding of ligand without leading to transactivation activity, due to the presence of co-repressors.

Ligands are chiefly considered to play central importance for PPAR-γ. This is either achieved by ligand-dependent or ligand-independent activity, which is conducted by co-activators and co-repressors, respectively [65]. Figure 5 illustrates the activation of PPAR-γ receptor through both ligand dependent and independent mechanisms. The ligand-dependent mechanism involves the activation of PPAR-γ after getting bound with ligand in LBD of C-terminus (AF-2), which later leads to the process of transcription. While, the ligand-independent mechanism is mainly due to N-terminus and kinase-dependent action, wherein ligands do not participate in the activation. Mu et al. have demonstrated the importance of ligand-independent action of PPAR-γ through N-terminus. They found that the activation rate of one sub-form, PPAR-γ2 was 5-fold greater than the other sub-form, PPAR-γ1 in adipose tissue via AF-1 activation domain, which suggests that there is a distinct activation profile between the two types [66]. These mechanisms provide a base for designing drugs. Further, the ligand-dependent mechanism comprises an agonist or antagonist mode of activation. The agonist mode may be full or partial depending on the structural differences and activation profile. Full agonist activity includes binding of ligand to the LBD of PPAR-γ that provides a flexible interaction higher affinity. This is carried out by folding of 12th helix together with helices H3 and H5 that leads to formation of a hydrophobic groove. H12 of LBD ought to define the activation efficiency through its stabilization or destabilization [67]. Therefore, binding of a ligand directly to helix 12 imparts a full agonist activity.

Previous structural studies have investigated that the potent synthetic ligand of PPAR-γ; TZDs have been seen to exert full agonist activity through such process. Mainly, TZDs have an acidic head group which tends to interact with intermolecular hydrogen bonds with amino-acid residues Ser289, His323, His449 and Tyr473 [68]. In contrast, partial agonist is characterized by indirectly interacting with H12by destabilizing it and lacks the intermolecular hydrogen bonds such as that of the full agonist with the four amino acids. However, the partial agonist stabilizes H3 and β-sheet region of LBD, which defines their activation efficiency. They are considered as weak activators of PPAR-γ compared with full agonists, which shows lower transactivation potential and imparts desirable effect [69]. Agonist-mediated activation of PPAR-γ has been reported to exhibit anti-inflammatory properties, growth inhibitory effects and prevent proliferation of many human cancer cell lines [34,64]. Likewise, the antagonist activity has also been observed to destabilize H12 and stabilize H3 and β-sheet. Thereafter, they inhibit co-activators binding and transcriptional activation [70]. Therefore, PPAR-γ antagonism is exploited to combat its aberrant expression. Numerous antagonists such as BADGE, PD068235, MK886, SR-202, GW6471, LG100641 and SR13904 have been identified [71]. Thus, the position of ligand binding within LBD constitutes central importance for PPAR-γ activation. At the core of this approach, research studies are targeting PPAR-γ and aiming to build up novel drug leads.

### 4.1. Endogenous Ligands

**Figure 6:**
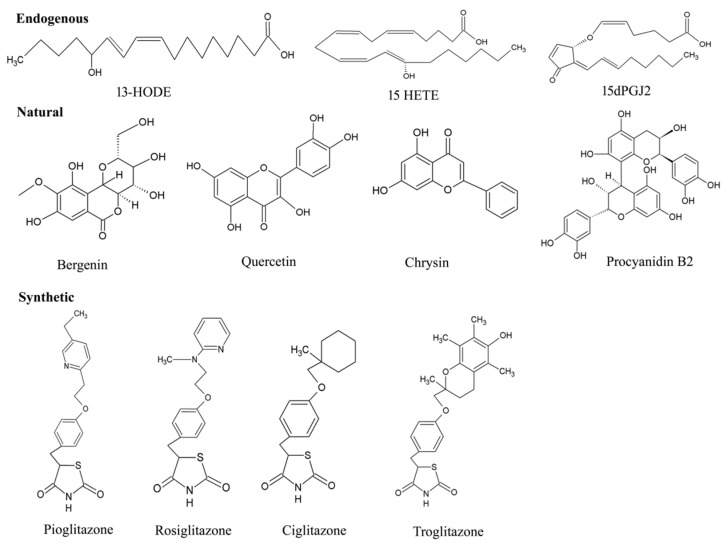
Chemical structures of various types PPAR-γ ligands (Endogenous, natural, and synthetic).

Due to the structural diversity of PPAR-γ, a variety of ligands bind to the protein receptor (Figure 6). Of note, owing to the above-mentioned mechanism of activation, even the ligands with weak efficacy can employ PPAR-γ driven effects. Endogenous and natural compounds are known as the weak agonists for PPAR-γ. The endogenous ligands constitute long chain polyunsaturated fatty acids (LC-PUFA) [72], J-series of prostaglandins [73] and arachidonic acids (15-Deoxy-∆-12,14-Prostaglandin J2-15d-PGJ2), eicosanoids, nitroalkanes, and oxidized phospholipids [74] including docosahexanoic acid (DHA). Compelling evidence indicates that these endogenous ligands can interfere with some hallmarks of cancer via PPAR-γ activation, which promotes growth inhibition, cell apoptosis, and resisting angiogenesis [75]. Substantial literature suggests that 15d-PGJ2 exerts various anti-inflammatory effects such as inhibiting cytokine expression, nuclear factor κB (NF-κB), and IkappaB (I-κB) kinase [76]. Zhao and colleagues demonstrated that the DNA binding activity of PPAR-γ significantly increased on intrastriatal injections on the locus of striatal hematoma. The compound markedly decreased NF-κB activation, prevented neutrophil infiltration and reduced cell apoptosis [77]. Given the central role of pro-inflammatory gene expression, low-density lipoprotein of PPAR-γ such as 9-hydroxyoctadecadienoicacid (HODE) and 13-HODE have also seen to attribute PPAR-γ activation [78]. Moreover, the endogenous ligands aids in low affinity for PPAR-γ, whereas high-affinity endogenous ligands are yet not known.

### 4.2. Natural Ligands

Certain natural products derived from plants have also appeared in the picture, which provides a promising pool of structures through their effective medicinal value for drug discovery. Traditionally, a wide range of medicinal plants have inspired the researchers to explore the mechanistic insights for PPAR-γ-activating potentiality. Vitamins, namely, tocopherol (α and γ), have been shown to serve as better modulator of PPAR-γ expression by upregulating in colon cancer cells [79]. A major group of antioxidants [80], herbs [81], fruits [82], seeds [83,84] and flowers [85] suggest protection against wide array of cancers. Some of the noteworthy examples for these natural products are extracts of *Maerua subcordata* (MS). The fruit, root, and seed extracts were revealed to up-regulate PPAR-γ expression level at 30g dry weight per liter (gDW/L), being non-cytotoxic in nature [86]. These plant natural products are known to have very fewer effects on the activation of PPAR-γ protein receptor.

According to epidemiological studies, some of the naturally occurring dietary flavonoid such as quercetin (3,5,7,3,4-pentahydroxyflavone), kaempferol (3,5,7,4tetrahydroxyflavone) and apigenin (4,5,7-trihydroxyflavone) also contribute in the activation process of PPAR-γ, which in turn are associated to the diminishing incidence of certain disorders: diabetes, coronary heart diseases and various types of cancer [87,88]. A study has implicated the efficiency of quercetin metabolites in stimulating PPAR-γ expression by inhibiting A549 cell growth with downregulation of cdk1, cyclin B and Matrix metallopeptidase 2 (MMP-2) expressions [87]. Quercetin-driven upregulation of PPAR-γ have fostered sufficient interest in generating several anti-cancer activities: promoting apoptosis, inducting cell cycle arrest and cell death in gastric, breast, lung, colon, prostate, and many other cancers [89]. Although there are only a few studies that have been able to demonstrate the efficacy of kaempferol in activating the protein receptor, PPAR-γ, it has gained attention for its anti-inflammatory and anti-cancerous effects [88]. Strikingly, Zhong et al. have illustrated the potentiality of natural flavonoid modulator, apigenin, in activating PPAR-γ by inducing apoptosis with cell cycle arrest at the G2/M phase in HCT-116, SW480, HT-29 and LoVo colon cancer cells, thereby upregulating pro-apoptotic proteins (NAG-1 and p53) and cell cycle inhibitor (p21) [90]. Unfortunately, despite such comprehensive evidence, these studies have been less convincing as the dietary flavonoids have offered fewer significant advantages in activating PPAR-γ, which pushes forth the search to uncover more novel agonists.

### 4.3. Synthetic Ligands

In addition to the endogenous and natural ligands, several synthetic ligands have been identified as strong agonists over the past few years. TZD were the first synthetic PPAR-γ agonists that were primarily designed to control T2DM by sensitizing insulin, which emerged as a major remedy for this disease. As a result, PPAR-γ is also known as the ‘glitazone receptor’ [91]. Although it comprises a diverse class of compounds, most importantly, rosiglitazone, troglitazone, ciglitazone and pioglitazone have been gaining a lot of attention towards anti-diabetic, anti-cancer, anti-microbial and anti-inflammatory activities [92,93,94]. Rosiglitazone represents the most potential compound amongst all TZDs, due to its superior pharmacokinetics and high affinity therapeutic value for cancer and inflammation. For instance, the anti-neoplastic effect of neuroblastoma cells (stromal (S) (SK-N-AS) and a neuroblast (N) (SH-SY5Y) phenotype) were deciphered, where Cellai et al. evaluated the efficacy of rosiglitazone for cell proliferation and invasion through the transactivation of PPAR-γ [95]. The findings addressed a significant reduction in proliferation and viability of cells by rosiglitazone, which relies on inhibiting cell adhesion and invasiveness in SK-NAS, but not in the SH-SY5Y neuroblastoma (NB) cell line. Interestingly, similar results were reported with a decrease in anti-apoptotic protein (Bcl-2) and an increase in pro-apoptotic protein (caspase 3) on human bladder cancer 5637 and T24 cell lines with the treatment of more than 10 µM concentration of rosiglitazone with PPAR-γ expression [96]. Further sensitization of breast cancer cell line, MDA-MB-231, predicted a mechanism by which rosiglitazone produces anti-tumor effects with a higher dose of 100 µM because it was required to transactivate a PPRE reporter construct for early changes in gene expression [97]. In addition, pioglitazone is under clinical trials for treatment of various diseases such as Alzheimer’s disease, CVD and diabetes. Recent research showed that administration of pioglitazone-loaded Poly (lactic-co-glycolic acid) delivery system on bleomycin-induced scleroderma model mice inhibited skin fibrosis within 60 min. Simultaneously, the researchers’ in vitro experiments revealed that pioglitazone reduced the migration ability and myofibroblast differentiation mediated by TGF-β in cultured fibroblasts [98]. Considerable interest has proved that along with troglitazone, ciglitazone has also been found to have an anti-cancer potential, owing to the property of chemopreventive agents. Ciglitazone induced cell cycle arrest at in the G1 phase in stomach cancer and suppressed cell growth [98]. Furthermore, it exerted a dose- and time-dependent anti-proliferative effect on A549 lung cancer cells both in vivo and in vitro, with significant upregulation of PPAR-γ expression [99]. Apart from the aforementioned TZDs, non-steroidal anti-inflammatory drugs (NSAIDs) were also discovered to exert stimulation for PPAR-γ but with low affinity. For example, indomethacin activated PPAR-γ in colorectal cancer with weak efficacy and did not result in anti-proliferative activity [100]. Other than this, sulindac sulfide, ibuprofen and diclofenacs activated PPAR-γ moderately [101]. Moreover, NSAIDs have been proven to reduce the risk of Alzheimer’s disease [63].

## 5. PPAR-γ Agonists in Various Diseases

### 5.1. Cancer

Cancer, one of the most frequent diseases worldwide, are characterized by continuous cell proliferation and dysregulation of the cell cycle. This outlines the importance of chemotherapeutic agents to the modulate cell cycle and/or apoptosis [102,103]. The approach for understanding means of PPAR-γ activation has gained considerable momentum in recent years which is found to be expressed in a variety of cancer cells. A vast amount of literature points to the fact that stimulation of PPAR-γ may be a key factor in producing various anti-cancer effects (Table 2). The PPAR-γ ligand, 15d-PGJ2, resists angiogenesis, promotes apoptosis, and inhibits migration [104]. A study accounted for apoptosis in colon cancer cells by 15d-PGJ2 via PPAR-γ activation by inhibiting telomerase activity and gene expression of human telomerase reverse transcriptase (hTERT) [105]. Numerous studies illustrated that 15d-PGJ2 may serve as an anti-cancer agent in oral squamous cell carcinoma cells [106] and gastric cancer [107] by promoting cell apoptosis. In addition, the protein receptor has been readily reported to induce apoptosis and inhibited proliferation of numerous other tumor cells [36,108,109]. Recently, a study illustrated chemopreventive nature of pioglitazone in a pre-clinical mouse model of squamous lung carcinoma. The authors have observed that pioglitazone prevented lung tumor development, reduced squamous lesions, and reduced that squamous dysplasia in an N-nitroso-trischloroethylurea (NTCU)-induced mouse model [110]. In one instance, rosiglitazone was to attributed an anti-fibrotic effect and inhibitory effect on paraquat (PQ)-induced acute pulmonary fibrosis in rats, administered with intraperitoneal injection of rosiglitazone [111]. Further, rosiglitazone was also observed to exert protective role in liver cancer cells by inducing apoptosis [112].

On the contrary, pioglitazone has also marked its therapeutic importance to combat cancer-associated pathological conditions. Recently, pioglitazone has been reported to overcome the effect of doxorubicin (DOX) resistance by modulating P-glycoprotein (P-gp) in a patient-derived orthotopicxenograft (PDOX) model of osteosarcoma. P-gp is well known to have a vital role in multidrug resistance (MDR) activity, which pumps out chemotherapy agents. In lieu of its significance, the study has broadened the application of pioglitazone on MDR in osteosarcoma treatment by successful activation of PPAR-γ [113]. One of the studies examined the importance of PPAR-γ activation with ERK1/2 accumulation in lung cancer cell line, NCI-H23, via mitochondrial pathway by inducing apoptosis on treating with troglitazone [93]. Considerable interest proved that along with troglitazone, ciglitazone has encouraged the observations on anti-cancer potential, owing to the property of chemopreventive agents. In one study, ciglitazone induced cell cycle arrest at G1 phase in stomach cancer, which suppressed cell growth [114]. Further, it exerted anti-proliferative effects on A549 lung cancer cells both in vivo and in vitro, with significant upregulation of PPAR-γ expression. With regard to this point, it is imperative to note that the TZD class of drugs has reduced the incidence of various cancers; however, to date, there is no such evidence on the potential impact of TZDs for active malignant disease.

### 5.2. Cardiovascular Disease (CVDs)

CVDs constitute a group of disease of the heart and blood vessels. These include ischemic stroke, arrhythmia, atherosclerosis, heart attack, heart failure and heart valve problems. The present lifestyle such as unhealthy diet habits, psychological stress and physical activity has collectively underlined the increasing mortality rate [115]. Nevertheless, muscles of heart, termed as myocardium, are the most vital part that surrounds and protects the heart. It requires a massive amount of energy (ATP production) in order to maintain the cardiac structure and function. Minor changes in flux are mediated by altering the substrate concentrations and allosteric modification of enzymes involved in these metabolic pathways. However, prolonged changes in cardiac metabolism are mediated at the gene transcriptional level. PPAR-γ has been shown to regulate the cardiac metabolism transcriptionally [116]. A study has documented the inhibition of cardiac hypertrophy, a condition resulting due to congestive heart failure through PPAR-γ dependent pathway in both in vivo and in vitro methods [8]. Another study demonstrated the efficacy of PPAR-γ natural agonist, quercetin, to impede the action of AP-1 protein in cardiac hypertrophy via PPAR-γ signaling. Quercetin lowered the blood pressure level and, remarkably, minimized the left-ventricular-to-body-weight (LVW/BW) ratio in hypertensive rats, while in vitro experiments suggested the suppression of transcription activity of AP-1 (c-fos, c-jun) protein (typically involved in cardiac hypertrophy) in H9C2 cells by PPAR-γ activation by quercetin [117]. Furthermore, PPAR-γ agonists have also been extensively reviewed for causing anti-inflammatory actions in ischemic stroke [118,119]. In a cohort study conducted between 2001 and 2013, it was observed that patients admitted due to ischemic stroke were potentially shown to be more cured with the administration of pioglitazone compared to the patients without pioglitazone. The recurrent ischemic stroke was prevented by pioglitazone [120]. Presently, pioglitazone is considered as a reliable cardioprotective agent due to its ability to reduce the risk of ischemic stroke and myocardial infarction without any direct harm on the myocardium [121]. On the contrary, there are studies where PPAR-γ can protect the cells from oxidative stress in oxidative stress-induced cardiomyocyte apoptosis by increasing the expression of Bcl-2 protein. Ren et al. explained the apoptotic effects induced by roziglitazone, wherein the ligand remarkably downregulated Bcl-2 protein in oxidative stress, H_2_O_2_-induced cardiomyocytes [122].

### 5.3. Type 2 Diabetes Mellitus (T2DM)

Diabetes has contributed massively as a public health problem in a global context. Intensive studies have reported that insulin resistance plays a vital role in the development of T2DM. This insulin is fueled by obesity which arises due to imbalanced lifestyle patterns and increased consumption of high-caloric food. These factors cause a decline in the response of pancreatic-β-cells that eventually develop resistance to increased insulin secretion. At this point, glucose intolerance and elevated levels of insulin in the body lead to T2DM [123]. Thus, hyperinsulinemia is known to regulate glucose metabolism that further overcome the insulin resistance. In addition, diabetes is also associated with other metabolic syndromes such as dyslipidemias, hypertension, and polycystic ovarian syndrome (PCOS). These are the prominent risk factors that underline the causes of T2DM [124]. For the past 30 years, PPAR-γ has been thought to serve as a significant target for the treatment of insulin resistance and T2DM [52]. There is evidence that attests to the fact that the activation of PPAR-γ induces insulin sensitization. The agonists of PPAR-γ are known to indirectly normalize the glucose profile by increasing the glucose uptake stimulated by the peripheral tissues and decreasing hepatic gluconeogenesis [125]. TZD merely aids in strong stimulation of PPAR-γ and improves the pharmacological treatment of T2DM (Table 2). Currently, TZD agonists for PPAR-γ are used therapeutically. Of note, they are considered as the second-line oral drug, which is sometimes administered alone or in combination with metformin, the first-line oral drug [126]. They are often known as insulin-sensitizing agents or anti-diabetic drugs. There are in vitro studies illustrating the binding potential of several TZD ligands with PPAR-γ, which connects well with in vivo affinity as insulin sensitizers [8]. On its activation, PPAR-γ pancreatic insulin secretion is found to decrease and reduces fatty acids in blood. Most of the effects of TZDs are driven by adipocyte differentiation, which increases glucose transporters (GLUT4) and induces lipogenic genes (AP2 and CD36) [127]. Reportedly, some derivatives of TZD, MSDC-0160 and MSDC-0602 were observed to cause anti-diabetic effects via PPAR-γ, mitochondrial membranes and the pyruvate carriers (MCP1 and MCP2) [6,128]. Based on these concepts, it is clear that PPAR-γ activation by TZDs accelerates the fibroblast differentiation process into adipocyte, which enhances GLUT4 expression and increases the insulin sensibility.

The members of TZD, namely, troglitazone, pioglitazone and rosiglitazone, were once approved for the treatment of T2DM. Evidence shows that troglitazone was the first TZD to be declared as an anti-diabetic drug due to its effective regulation of glycemia. Unfortunately, it was discontinued from the market because of serious liver toxicity which was reported in 100,000 patients [129]. On the other hand, pioglitazone and rosiglitazone were also licensed for controlling hyperglycemia in T2DM, but they have been also removed from the market. Treatment of pioglitazone proved to increase body weight in an in vivo study wherein 48 volunteers with T2DM were subjected to pioglitazone (30 mg/day) or 12 weeks with placebo. The study found that pioglitazone was associated with increased expression of genes in glycerol-3-phosphate synthesis, adipocytes, c-Cbl-associated protein, tumor necrosis factor-alpha, angiopoietin-like 4, leptin, resistin, and 11-beta-hydroxysteroid dehydrogenase type 1 via the activation of PPAR-γ [130].

There are various small molecules that act as selective PPAR-γ agonists and are reported to elicit anti-diabetic effects. For example, 30 μM of F12016 has been shown to selectively activate PPAR-γ and no other isoforms. It has been further characterized for possessing glucose-lowering and insulin-sensitizing properties in diabetic KK-Ay mice [131]. The compound remarkably increased glucose uptake and obstructed phosphorylation mediated by cyclin-dependent kinase [132]. In contrast, some of the endogenous agonists such as 13-hidroxioctadecanoic acid (13-HODE) and 15-hidroxieicosatetraenoic acid (15-HODE) and prostaglandins of the A, D, and J series, which are low-density lipoproteins, are also recognized as anti-diabetic agents. In addition, natural agonists of PPAR-γ have also been reported to improve disorders related to diabetes such as glycolipid metabolism and obesity. Pan et al. confirmed the underlying mechanism, a bioactive compound, curcumin, in glycolipid metabolism. In their in vivo experiments in male C57BL/6 J obese mice for eight weeks, curcumin displayed diminished activities of body weight, serum lipid profiles and fat mass with a concomitant increase in the insulin sensitivity via activation of PPAR-γ. Meanwhile, in their in vitro experiments in 3T3-L1 adipocytes, curcumin decreased glycerol release and elevated the uptake of glucose through stimulation of PPAR-γ and C/EBP-α [133]. Altogether, PPAR-γ agonists have the ability to regulate gene expression in diabetes and its related disorders.

### 5.4. Autoimmune Diseases (AIDs)

AIDs is a condition that occurs in an immune system which is characterized by prolonged inflammatory reaction with the production of auto-antibodies and loss of self-tolerance or immune tolerance. AIDs are categorized into organ-specific diseases, such as rheumatoid arthritis and autoimmune thyroid diseases, and systemic diseases, such as systemic lupus erythematosus and systemic sclerosis [134]. As we know that PPAR-γ agonists exert anti-inflammatory responses, the molecules participating in immune feedback are considered as potential therapeutic targets for its treatment.Recent studies have exemplified that PPAR-γ agonists also exert a protective role in AIDs (Table 2). One group suggested the upregulation of microRNA (miR)-124 by PPAR-γ in their in vitro and in vivo work. They found that the elevation of miR-124 could attenuate the generation of pro-inflammatory cytokines and augment the expression of miR-142-3p. This was in turn observed to inhibit pro-inflammatory mediator high-mobility group box-1 (HMGB1) expression, which is normally found to be increased in AIDs [7]. Another group demonstrated the development of autoimmune kidney disease, glomerulonephritis, in mice lacking macrophage-specific PPAR-γ or RXR-α, which led to the production of auto-antibodies to nuclear antigens. The lack of PPAR-γ or RXR-α manifested a deficiency in phagocytosis, loss of immune tolerance, and clearance of apoptotic cells in the mice [135].

The activation of PPAR-γ has been also reported to persuade the macrophage polarization towards an immune-modulatory M2-like phenotype that ultimately reduces neutrophil migration [136]. Cheng et al. showed that the activation of PPAR-γ by pioglitazone diminished TNF-α-induced TGF-β, hyaluronan (HA), and HAS3 expressions substantially in the active stage patients with Graves’ ophthalmopathy (GO) over normal controls [137]. It has been proven that some of the potent PPAR-γ agonists such as ziglitazone, pioglitazone and GW347845 diminished the proliferation of T-cell and production of IFN-γ, TNF-α and cytokine [138]. Studies have also attested that continuous activation of PPAR-γ can prevent Th17 differentiation in murine CD4+ T cells and human models. Further, IL-17 expression is weakened, and the release of inflammatory cytokines is decreased [139]. Moreover, it has been elucidated that PPAR-γ ligands can lead to synovial cell apoptosis. For instance, for the maintenance of rheumatoid synovitis, a significant transcription factor, NF-κB, is required, and the activation of fibroblast-like synoviocytes (FLSs) with PPAR-γ can impede the pro-inflammatory activity of NF-κB [140]. In addition, a group has established inhibition of inflammation in the lupus-prone mouse model with primary biliary cirrhosis-like cholangitis via PPAR-γ activation by 15d-PGJ2 [141]. It appears that curcumin, a bioactive compound, effectively suppresses the autoimmune response by decreasing the activity of pro-inflammatory interleukins and cytokines. Bernardo et al. discerned that curcumin enhanced the differentiation of oligodendrocyte progenitor and inhibited the arrest of maturation in them via PPAR-γ activation [142]. These studies laid the foundation for PPAR-γ agonists to be promising in various AIDs.

### 5.5. Inflammatory Diseases

Inflammatory diseases emerge in the central nervous system (CNS), and the devastating effects include nerve damage, inflammation of CNS, loss of vision, fatigue, pain, demyelination and impaired coordination. The critical role of PPAR-γ agonists in modulating immune responses has been established and extensively documented [63,143] (Table 2). The agonists have the ability to inhibit the activated microglia, manage inflammation and defend the neurons from various degenerative diseases of CNS such as Parkinson’s disease, multiple sclerosis and Alzheimer’s disease [144]. It was observed that PPAR-γ agonists have key role in suppressing the activation of macrophage or monocyte lineages [145]. A computational study screened a natural product library that revealed a total of potent 29 agonists for PPAR-γ. These agonists were further carried for in vitro analysis wherein six flavonoids were detected to stimulate transcriptional activity of PPAR-γ in THP-1 macrophages. Among these, psi-baptigenin was observed to be the most potent agonist with an EC50 of 2.9 μM [146]. Xu et al. identified an endogenous ligand, 25-hydroxycholesterol-3-sulfate (25HC3S) to activate PPAR-γ in human macrophages. 25HC3S is an oxysterol that has key role in regulating lipid homeostasis and metabolism. The authors found that this cholesterol metabolite, 25HC3S markedly elevated the levels of nuclear PPAR-γ with a decrease in NF-κB protein levels [147]. Therefore, these observations suggest that PPAR-γ agonists have the ability to inhibit various transcription factors of immune response such as Signal transducer and activator of transcription 1 (STAT-1), NF-κB and activator protein 1 (AP-1), which in turn impedes their gene expression. A pioneering work by Glass and co-workers reported the molecular mechanisms that regulate transrepression of NF-κB responsive genes. Their work showed SUMOylation of PPAR-γ upon binding of PPAR-γ ligands with SUMO1 via NCoRco-repressor. Although the process of SUMOylation seems to take place in NF-κB-activating stimuli, it tends to maintain the responsive genes of promoter region in repressed state [148,149].

In addition, the PPAR-γ endogenous agonist, 15d-PGJ2, also acts to restrict the degradation of I-κB by inhibiting I-κB kinase activation [76]. Further, 15d-PGJ2 has also been documented to inhibit the binding of NF-κB to its DNA-response elements [77]. The effects of 15d-PGJ2 on immune function were first described by Petrova et al. The authors explained the inhibitory effect of 15d-PGJ2 of LPS induction in murine BV-2 microglial cell line for NO and iNOS expression. In comparison, the potent synthetic PPAR-γ agonist failed to suppress the LPS induction [150]. In addition, Cuzzocrea et al. illustrated the potency of synthetic agonist, rosiglitazone, which elicited various anti-inflammatory effects in Carrageenan rat paw oedema model such as formation of pleural exudate, attenuation of paw oedema, mononuclear cell infiltration and histological injury. Thus, rosiglitazone led to a substantial decrease in acute inflammation in the rat [151]. PPAR-γ activation plays a key role in suppressing various gene expressions inflammatory responses. In an instance, rosiglitazone has been shown to repress the transcription of Fractalkine receptor gene via activation of PPAR-γ. Fractalkine receptors potentially regulate leukocyte adhesion and migration in immune responses to inflamed peripheral tissues. In addition, PPAR-γ activation also led to the inhibition of nuclear export of Fractalkine in endothelial cells and, thus, prevented the translocation of Fractalkine receptor [152]. From this point of view, PPAR-γ agonist offers a new angle in the pharmacologic management of various inflammatory diseases as well.

### 5.6. Dermatological Diseases (DDs)

Human epidermis and hair follicles (HFs) are found to be expressed by all three PPAR isoforms. Most of the prominently differentiated stratum basal keratinocytes in the epidermis contain PPAR-γ. The basal layer of hair cuticle, outer root sheath, cortex and connective tissue sheath are all shown to express PPAR-α, β/δ and γ in the HF [153]. However, only PPAR-γ and PPAR-β/δ are expressed in the inner root sheath keratinocytes. PPAR-γ expression decreases with terminal sebaceous differentiation in human sebaceous glands (SGs), with substantial expression in basal and early developed sebocytes. During puberty, PPAR-γ is the only one which is expressed more significantly in sebocytes [154]. Numerous inflammatory mediators and cytokines are produced by many different cell types, including macrophages, epithelium, smooth muscle cells, endothelium dendritic cells, and lymphocytes, which have been found to be inhibited by certain PPAR-γ ligands. By opposing the actions of transcription factors such as those in the NF-κB family, PPAR-γ directly controls the expression of pro-inflammatory genes in a ligand-dependent way [149]. Transrepression is a key mechanism that explains how PPARs can obstruct the functions of these transcription factors. Furthermore, PPAR-γ reduces the production of adhesion molecules and inhibits Langerhans cell functions. Based on its anti-inflammatory properties, PPAR-γ represents a significant research target for the comprehension and management of numerous DDs [155]. Numerous studies have also shown that TZDs have a number of additional and possibly significant effects on the structure and function of the skin, such as promoting keratinocyte differentiation, reducing inflammation, and enhancing permeability barrier cellular homeostasis, which has led to their use in the treatment of various skin pathologies [156]. However, the widespread use of TZDs has been restricted due to the drugs’ potential side effects, some of which may be life-threatening. As a result, the researchers are focusing on creating new classes of partial and efficient PPAR-γ modulators that maintain the anti-inflammatory action of its agonists while minimizing their negative side effects [154].

**Table 2:** List of ligands activating PPAR-γ and their significant role in various diseases.

Type of PPAR-γ Ligand	Name of the Ligand	Source	Disease	Effect in Disease	References
Endogenous	13-HODE *	n-3 LC-PUFA *	Cancer	Anti-proliferative activity, cell cycle arrest (G1) and apoptosis	[157]
			Multiple sclerosis	Reduced clinical severity of allergic encephalomyelitis	[158]
	15-HETE *	n-3 LC-PUFA	Cancer	Anti-proliferative activity, cell cycle arrest (G1), and apoptosis	[159]
			CVD	Anti-platelet and anti-thrombotic effects	[160]
	15d-PGJ2*	Prostaglandin J2 derivative	Cancer	Cell cycle arrest, apoptosis and reducing ornithine decarboxylase activity	[161]
			Inflammatory disorders	Regulates expression of surface proteins, T-cell activation, and related inflammatory cytokines	[162]
			AID	Anti-inflammatory effects in primary biliary cirrhosis patients;	[141]
			Asthma	Inhibited T(H)2 type cytokine IL-5production	[163]
Natural	Procyanidin B2	Flavonoid	Hepatic diseases	Inhibited nicotine-induced pyroptosis	[164]
	Artepillin C	*Baccharisdracunculifolia*	T2DM *	Induced adipocyte differentiation and glucose uptake	[165]
	Lectins and viscotoxins	Herbs-*Viscum album* L.	Cancer	Apoptosis, inhibition of angiogenesis	[166]
	Bergenin	Herb of *Saxifragastolonifera* Curt.	Inflammatory disorders	Alleviated disease symptoms of dextran sulfate sodium (DSS)-induced colitis	[81]
			Asthma	Prevented GLS1-dependent glutaminolysis	[167]
	Antioxidants (Ascorbic acid and phytochemicals)	Whole-apple extracts	Cancer	Inhibition of tumor-cell proliferation in prostate and breast cancer	[82]
	1,1-Bis(3′-indolyl)-1-(*p*-trifluoromethylphenyl)methane	*p*-substituted phenyl analogues	Cancer	Cell cycle arrest (G_0_/G_1_-S) in endometrial cancer	[168]
	Chrysin	Flavonoid	Asthma	Alleviated ovalbumin-airway hyperresponsiveness	[169]
	Quercetin	Flavonoid	Cancer	Tumor-inhibitory effects in breast cancer	[170]
			Cancer	Anti-proliferative and anti-migratory effects in lung cancer	[171]
	CucurbitaneTriterpenoid	Extract of wild bitter gourd (*Momordicacharantia*)	Cancer	Anti-proliferative effect induced apoptotic death in breast cancer cells	[172]
			Insulin resistance	Induced adipocyte differentiation and glucose uptake	[173]
			T2DM	Induced glucose uptake	[174]
	Methanolic extract *Pterocarpus marsupium*	isoflavone	T2DM	Induced glucose uptake and elevated Glut-4	[175]
Synthetic	Pioglitazone	TZD *	Neurological disease	Inhibited mTOR activation and prevented increase in IL-1β and IL-6.	[176]
			Neurological disease	Reduced hyperalgesia and astrocyte activation	[177]
			Psoriatic Arthritis Response	Inhibited angiogenesis and suppressed pro-inflammatory cytokines	[178]
			Asthma	Reduced regulator of G protein 4	[179]
			SRD	Inhibited bleomycin-induced skin fibrosis	[98]
	Rosiglitazone	TZD	Ischemia Stroke	Limited postischemic injury in normal and diabetic hearts	[180]
			Asthma	Reduced bronchial inflammation	[181]
			Allergy	Decreased ROS generation, expression of T(H)2 cell cytokines in lungs after ovalbumin inhalation	[182]
	Ciglitazone	TZD	Cancer	Inhibitory effects on lung cancer	[183]
			Cancer	Inhibitory effects on prostate cancer	[184]
	Troglitazone	TZD	Cancer	Reduced c-Myc levels in prostate cancer	[185]
			Psoriasis	Inhibited proliferation of psoriatic human keratinocytes	[186]
	GW347845	Non-TZD	AID	Anti-inflammatory and anti-proliferative effects	[138]

* 13-HODE-13-Hydroxyoctadecadienoic acid; n-3 LC-PUFA-n-3 long chain polyunsaturated fatty acids; 15-HETE-15-hydroxyeicosatetraenoic acid; 15d-PGJ2-15-Deoxy-∆-12,14-Prostaglandin J2; T2DM, Type 2 diabetes mellitus; TZD, Thiazolidinedione.

## 6. Challenges Faced and Knowledge Gaps for PPAR-γ Agonists

There has been controversy over many endogenous ligands due to their uncertain intracellular levels and interaction with PPAR-γ, which is biologically insignificant [187]. Thus, they activate PPAR-γ with relatively low affinity, i.e., they act as weak agonists. On the other hand, the natural compounds have some concerns about their limited efficiency, poor bioavailability, inadequate absorption, inappropriate solubility, and non-specificity [188]. Further, increasing evidence accounts for the adverse effects of the potent PPAR-γ agonist, TZDs. TZDs have been reported as powerful activators of PPAR-γ, ultimately showing profound anti-inflammatory, anti-cancer, and anti-diabetic activities. However, the use of TZDs have been withdrawn from market due to higher risk in cardiovascular, heart failure, weight gain, edema, sodium retention and decreased glucosuria [189,190]. Although TZDs exhibit various activities, the class of drugs has been involved for increased risk. Prolonged usage of TZDs has been reported to increase the level of PPAR-γ expression in bladder cancer [191]. According to investigations, rosiglitazone seems to be associated with a lower risk of thyroid cancer [192] and breast cancer [193], whereas it brings a higher risk of bladder cancer [194], due to which it has been banned in Europe. The first TZD to be stopped from the market of USA as well as UK was troglitazone, in the year 1999 and 2000, due to its major hepatotoxicity activities [195].

It is worth noting that a study conducted by Nissen et al. reported that rosiglitazone was shown to have a 1.4-fold increased risk of acute myocardial infarction (AMI) in the year 2007 [196]. In particular, the incidence rates of heart failure, strokes and death was observed for the patients when administered with pioglitazone for long-term treatment [94,197]. In addition, ciglitazone has been never approved for medication of its weak clinical activity, though it serves as a prototype for all TZDs. Moreover, it is also reported that an elevated level of TZDs tends to cause visceral fat accumulation, which contributes to major weight gain [190] and increased sodium, and poor water reabsorption in kidney, which leads to fluid retention, ultimately causing edema. Further consequences include fluid retention and weight gain, which lead to congestive heart failure [198]. TZDs are therefore restricted due to concerns for their adverse side-effects, which has weakened their preventive measures in cancer therapies as well as in diabetes treatment. As it acts as a full agonist for PPAR-γ, studies have shown it to express the protein receptor more than its optimized level, which leads to over-activation of PPAR-γ, eventually leading to tumor progression. Hence, proper examination is required to select the exact ligand for PPAR-γ, because natural and endogenous ligands serve as weak agonists, whereas synthetic ligands such as TZDs act as full agonist for PPAR-γ. A selective measure is necessary to re-explore the agonists which will have anti-inflammatory, anti-diabetic and anti-cancer characteristics, sans toxicity and akin to the proper activation of PPAR-γ.

## 7. Significance of PPAR-γ Partial Agonist

The compounds that stimulate PPAR-γ in a desired manner, i.e., lesser effect than synthetic agonists and greater effect than weak agonists, are referred to as partial agonist. One such example is selective PPAR-γ modulators (SPPAR-γMs), which were built to reduce the unwanted side-effects of synthetic agonists by optimizing the gene expression signature [199]. A natural product named amorfrutins, were observed to act as SPPAR-γM that exerted decreased gene expression in the fat storage process compared to the synthetic agonist, rosiglitazone [200]. Amorfrutin is a low nanomolar-binding2-hydroxy benzoic acid derivative of salicylic acids that promoted anti-inflammatory and insulin-sensitizing effects in diabetes mouse models. Notably, some of the synthesized derivatives of amorfrutin from a common building block library served as PPAR-γ partial agonists and promising anti-diabetic drugs [201]. Besides SPPAR-γM, telmisartan is angiotensin receptor blocker which also aids in partial activation of PPAR-γ. The compound was noticed to exert noteworthy effects against inflammatory responses, oxidative stress and EMT [202]. A group demonstrated the counteraction of TGF-β1-induced EMT in human renal proximal tubular epithelial (HK-2) cells through the partial activation of PPAR-γ [203]. Similarly, another study suggested that Treatment of telmisartan in HK2 cells inhibited EMT induction via PPAR-γ-AKT/STAT3/p38 MAPK-Snail pathway in vitro and in vivo by oxalate and calcium oxalate crystals [204]. In contrast, it has been demonstrated that flavonoids extracted from lemon balm (*Melissa officinalis*) [205] and chamomile (*Matricaria chamomilla*/*Matricaria recutita*) [80] flowers behave as a partial agonist for PPAR-γ by activating it with a half-maximal effective concentration (EC50) of 86 mg/mL and had 26% of maximal potency as compared to rosiglitazone (TZD). According to epidemiological studies some of the naturally occurring dietary flavonoids such as quercetin (3,5,7,3,4-pentahydroxyflavone), Kaempferol (3,5,7,4tetrahydroxyflavone) and apigenin (4,5,7-trihydroxyflavone) also contribute to the activation process of PPAR-γ which in turn are associated with a diminishing incidence of various types of cancer [87,88]. These studies certainly prove that the biological function and mechanism of PPAR-γ could improve the rationale of ligand development. Targeting various molecules to the ligand binding region of PPAR-γ can anticipate selective modulation. This would result in greater expression and a promising pharmacological approach.

## 8. PPAR-γ Partial Agonists Involved in Post-Transcriptional Modification and Disease-Fate Decision

It has been discovered that partial agonists are neoteric substances that have a substantial affinity for PPAR-γ and display the effects that cause insulin sensitivity, anti-cancer and anti-inflammatory characteristics, and rescue various heart disease. The beneficial effects of PPAR-γ partial agonists in various diseases are summarized in Table 3. These ligands cannot completely saturate PPAR-γ activity; nevertheless, they lessen the negative effects of TZDs. Zheng and colleagues virtually screened a library of compounds using AlphaScreen assay and found ionomycin, an antibiotic to act as partial agonist for PPAR-γ. Their basic aim was to develop alternative and better PPAR-γ ligands than TZDs, which are reported for their severe side effects. Ionomycin showed distinctly interacted with PPAR-γ LBD over the TZDs and improved hyperglycemia and insulin resistance in a mouse model of diabetes. Further in vitro and in vivo experiments showed the inhibition of PPAR-γ phosphorylation at Ser273 by cyclin-dependent kinase 5 [206]. The partial agonists also constitute SPPAR-γMs, NSAID and non-TZD partial agonist (nTZDpa) [207]. Some of these SPPAR-γMs share an indole moiety with NSAIDS. Schug and collaborators proved that the indole containing non-TZD partial agonist (nTZDpa) enhance insulin sensitivity in obese mice while reducing unfavorable effects on weight gain, adiposity, and cardiovascular hypertrophy [208]. Similarly, NSAID compounds have undergone in vitro testing as partial PPAR-γ agonists, followed by pharmacokinetic studies in rats and in insulin-resistant mice models. These are benzoyl 2-methyl indoles, often known as carboxylic acid indoles. A PPAR-γ modulator termed as SPPARM5 functioned as a partial agonist of PPAR-γ, with some reduction in the ability to promote adipose gene expression, while retaining the insulin sensitizing capabilities [209]. SPPARM5 was also examined in Zucker rats in contrast to rosiglitazone for effects on plasma and extracellular volume, heart weight, and fluid retention [207].

The PPAR-γ activation by partial agonists controls a variety of parameters, including protein expression levels, ligands, and transcriptional cofactors. These factors eventually influence the course of the disease, thus helping in determining the disease fate. Post-transcriptional modifications (PTMs) following PPAR-γ activation have the ability to modify protein shape, control protein interactions, and change the moiety between receptors and ligands, all of which affect how transcription of downstream genes is regulated [210]. The key PTMs which influence the course of development of a disease are highlighted as follows:

### 8.1. Phosphorylation

PPAR-γ could be phosphorylated at various locations with various stimuli, leading to various biological effects [211]. Ser273 (Ser245 in isoform 1) and Ser112 (Ser82 in isoform 1) are the primary sites for PPAR-γ phosphorylation by cyclin-dependent kinase (Cdk) and MAPK. As a result of PPAR-γ S273 being phosphorylated by Cdk5, less adipogenesis and transcriptional activity is generated [212]. TNF-α, IL-1β, and other inflammatory cytokines can be expressed as a result of PPAR-γ phosphorylation, which can also encourage the growth of foam cells and hasten the progression of atherosclerosis [213]. In response to exposure to chemicals that damage DNA, cancer cells phosphorylate the PPAR-γ Ser273 protein. Genetically or pharmacologically inhibiting this phosphorylation causes a build-up of DNA damage that leads to apoptotic cell death. Furthermore, p53 signaling is deregulated when PPAR-γ phosphorylation is inhibited, and biochemical studies demonstrate that PPAR physically interacts with p53 that is dependent on Ser273 phosphorylation [202]. These findings suggest that PPAR-γ plays a vital role in regulating the p53 response to cytotoxic therapy, which can be controlled for therapeutic benefits.

### 8.2. SUMOylation

The SUMOylation of PPAR-γ is termed as transrepression. According to Ying et al. cellular inflammation brought about by lipopolysaccharide is suppressed by SUMOylation of PPAR-γ by a partial agonist, which inhibits NF-κB [210]. The SUMOylation pathway is composed of the proteases namely SUMO E1, E2, and E3, which can change the way that target proteins are regulated in transcription. This mechanism is extremely important for controlling the course of the cell cycle and the tumorigenic processes [214]. Therefore, targeting SUMOylation of PPAR-γ can provide a promising solution to determine disease fate and, hence, their potential cure [215]. According to Phan et al., SUMOylation of PPAR-γ links lipid metabolism to its tumor-suppressive properties in lung cancer. They discovered that both in vitro and in vivo, PPAR-γ ligand activation significantly increased de novo lipid production as well as fatty acid beta (β)-oxidation in lung cancer [216]. More significantly, it transpires that SUMOylation of PPAR-γ was necessary for regulation of lipid metabolism. More in-depth biochemical research showed that PPAR-γ-mediated lipid production degrades nicotinamide adenine dinucleotide phosphate (NADPH), which raises the quantity of reactive oxygen species (ROS) in the mitochondria and disrupts the equilibrium of REDOX reactions in lung cancer [217]. As a result, liganded PPAR-γ SUMOylation is essential for cellular lipid metabolism as well as for inducing oxidative stress, which helps PPAR-γ act as a tumor suppressor. This study provides crucial insight into future translational and clinical research into addressing PPAR-γ control of lipid metabolism in lung cancer patients with T2DM.

### 8.3. Ubiquitination

Ubiquitination modification of PPAR-γ not only controls the proteasome-mediated destruction of target proteins but also functions as a “scaffold” to draw in more proteins to form signal complexes. When PPAR-γ binds to the selective ligand, it experiences substantial conformational changes. PPAR-γ is targeted for proteasomal degradation by the PPAR E3 ligases Makorin RING finger protein 1 (MKRN1) [218] and seven in absentia homolog 2 (SIAH2) [219]. Alternatively, it can encourage proteasome-dependent disassembly and bind with ubiquitination-associated enzymes, thus negatively affecting its overall transcriptional activity [215]. An E3 ubiquitin ligase, namely, neural precursor cell expressed developmentally down-regulated protein 4 (NEDD4) interacted with the hinge and LBD of PPAR-γ. Further, it underwent ubiquitination of PPAR-γ in adipocytes, as reported by Carvalho et al. [220]. The E3 ubiquitin ligase tripartite motif containing 23 (TRIM23) promotes PPAR-γ stability by inhibiting its proteasomal degradation and controlling adipocyte development. This could thus provide a potential solution to trace various diseases which involve adipogenesis dysregulation [221]. In clinical research, TZDs activators (used to treat diabetes) and the proteasome inhibitor Bortezomib (used to treat cancer) have both been used to pharmacologically control the PPAR-γ and the ubiquitin proteasome system [222]. The development of medications for the treatment of colorectal cancer may be attracted to a combination used in order to activate the transcription factor at least twice. It would be crucial to identify different cancer subtypes that, as a result of particular molecular abnormalities, may be especially vulnerable to PPAR-γ ubiquitination.

**Table 3:** Effect of PPAR-γ partial agonists in disease-fate decision.

PPAR-γ Partial Agonist	Type of Compound	Disease	Effect in Disease	References
SPPAR-γM5	SPPAR-γM *	T2DM	Reduced the insulin resistance index	[69]
PAR-1622 *	SPPAR-γM	T2DM	Induced adipocyte differentiation and improved hyperglycemia	[214]
PAM-1616 *	SPPAR-γM	T2DM	Improved hyperglycemia	[223]
FK614 *	SPPAR-γM	T2DM	Reduced the insulin resistance index	[224]
F12016 *	SPPAR-γM	T2DM	Insulin-sensitizing and glucose-lowering properties	[132]
KDT501 *	Chemically derived from substituted 1,3-cyclopentadione	Inflammatory disorders	Anti-inflammatory effects in monocytes/macrophages	[225]
GQ-16 *	TZD-Derived	Obesity	Reduced high fat diet-induced weight gain	[211]
		Cancer	Anti-proliferative effects in breast cancer	[212]
Telmisartan	Angiotensin type 1 receptor blocker	Inflammatory disorders	cerebroprotective effect	[213]
		T2DM	Ameliorated vascular endothelial dysfunction and protected against diabetic vascular complications	[202]

* SPPAR-γM-selective PPAR-γ modulators; PAR-1622-(S)-2-ethoxy-3(4-(5-(4-(5-(methoxymethyl)isoxazol-3-yl)phenyl)-3-methylthiophen-2-yl)methoxy)phenyl)propanoic Acid; PAM-1616-(S)-2-ethoxy-3-(4-((3-methyl-5-(4-(3-methylisoxazol-5-yl) phenyl) thiophen-2-yl) methoxy) phenyl) propanoic acid; FK614-3-(2,4-dichlorobenzyl)-2-methyl-N-(pentylsulfonyl)-3-Hbenzimidazole-5-carboxamide; F12016-2-[2-(1,2-dimethyl-1H-indol-3-yl)-2-oxo-acetylamino]-benzamide; KDT501-Potassium salt of the n-(isobutyl) congener of a tetrahydro iso-α acid; GQ-16-(5Z)-5-(5-bromo-2-methoxy-benzylidene)-3-(4-methyl-benzyl)-thiazolidine-2,4-dione.

## 9. PPAR-γ Partial Agonists in Cancer Therapeutics

As already discussed in the above sections that inflammation and immunity are strongly regulated by PPAR-γ, suggesting being useful in cancer immunotherapy. The activation of PPAR-γ may activate many signaling pathways in anti-tumorigenic activity. According to Zhao and collaborators, myeloid-derived suppressor cells (MDSCs) infiltrate PPAR-γ attenuated mouse melanoma cells and caused generalized non-specific inflammatory reactions [214]. In addition, PPAR-γ ligand binding interaction results in a positive effect leading to prevention of tumor growth (Table 4). This positive action is accomplished via preventing the overproduction of reactive oxygen species (ROS) by MDSCs, mTOR pathway, and an additional receptor for advanced glycation end products (RAGE pathway) which work in conjunction [64,215]. Macrophages are highly diverse and plastic. In terms of malignancy, tumor-associated macrophages are particularly prevalent and pro-proliferative within tumors. They promote tumor growth and spread by suppressing the immune system and promoting angiogenesis. When activated particularly by a partial agonist, PPAR-γ may decrease the release of M1 pro-inflammatory and pro-tumor M2-cytokines without changing macrophage polarization, thus producing anti-tumor effects [215,217]. When administered as monotherapy, TZDs provide a significant clinical anti-cancer action in the majority of trials, but they also have potentially fatal adverse effects. As a result, the researchers are now trying to test partial agonists rather than the synthetic TZDs in order to sequester positive cancer therapeutic results [218].

Telmisartan is a partial PPAR-γ agonist that was noticed to exert noteworthy effects against inflammatory responses, oxidative stress and EMT [204]. Deoxyelephantopin (ESD), another PPAR-γ partial agonist, was reported to potentiate apoptosis, inhibit invasion, and abolish osteoclastogenesis by Zou et al. [69]. It was isolated from the Wild plant of *Elephantopus carolinianus* and exhibits anti-tumor, anti-inflammatory, and invasion-inhibiting activities. Their findings demonstrated that ESD-induced PPAR-γ knockdown could cause HeLa cell line death and cell cycle arrest during G2/M phase in a dose-dependent manner [219]. Gan and associates in their investigation revealed Tetrazanbigen (TNBG), a new sterol isoquinoline derivative with poor water solubility that produced mild inhibitory effects on human tumor cell lines via lipoapoptosis induction [220]. The primary goal of employing TNBG as a PPAR gamma partial agonist was to cause tumor cells to undergo lipoapoptosis. The underlying theory demonstrates how excessive lipid accumulation interferes with cancer cells ability to use lipids and how an unrestricted accumulation of lipid droplets would take up most of the cytoplasm, impair the operation of other organelles, and eventually cause differentiated cancer cells to undergo lipoapoptosis [221].

## 10. PPAR-γ Partial Agonists under Clinical Trials

Although various PPAR-γ partial agonists are now undergoing clinical studies, the partial agonists identified to date have not yet acquired FDA approval. INT131 (formerly AMG-131), which has advanced through Phase II clinical trials, is the most well-known and maybe most promising example [228]. Netoglitazone, Balaglitazone, Metaglidasen, and Halofenate are further PPAR-γ partial agonists that have progressed to or finished Phase II clinical trials [229,230,231]. When compared to rosiglitazone, partial agonists of PPAR-γ can exhibit a broad spectrum of transcriptional activation [229]. In adipocyte cell models such 3T3-L1 mouse fibroblast cells, partial agonists have demonstrated effects that cause insulin sensitization but have not been shown to cause fatty acid accumulation. Beyond their functional efficacy, the exact atomic and molecular characteristics of PPAR-γ partial agonists are still unknown.

A novel partial PPAR-γ agonist called balaglitazone was introduced by Dr. Reddy’s laboratory in India as a cure for T2DM. Balaglitazone dramatically lowers HbA1c levels and has a better safety profile than full agonists, since it is a selective partial PPAR-γ agonist. As a supplement to insulin therapy, balaglitazone offers strong glycemic control, and phase trials revealed a tendency towards fewer severe side effects. However, because of unfavorable side effects such inflammation, ROS production, and altered gene expression, the investment was stopped in 2011 [231]. Murakami et al. reported Efatutazone, an oral, highly selective PPAR-γ agonist, is superior to second-generation TZDs such as pioglitazone and rosiglitazone in terms of effectiveness [232]. Efatutazone suppressed the growth of human anaplastic thyroid and pancreatic tumor cell lines in pre-clinical tumor models, as well as human colorectal and anaplastic thyroid tumor cell xenografts in nude mice. Efatutazone demonstrated good safety, tolerability, and disease control at dosages of 0.10–1.15 mg bis in die (bid) in a phase I study in patients with advanced solid tumors [233]. A recent phase I clinical trial using efatutazone plus paclitaxel in patients with advanced anaplastic thyroid carcinoma revealed similar positive safety results, disease control, and disease stability [234]. These phase I clinical studies proved that Efatutazone can be used alone or in combination with other chemotherapeutic drugs as a novel approach for the treatment of advanced metastatic tumors, as a result of its unique property as a highly selective PPAR-γ activator. Additionally, this might offer useful information about the therapeutic implications of selective PPAR-γ activation in the regulation of carcinogenesis.

Such clinical studies have thus proven that potential PPAR-γ partial agonists can be worked upon and implemented into the therapeutic lines of cancer and are expected to yield better outcomes than the synthetic ligands such as TZDs. Although none of the PPAR-γ partial agonists have yet been approved by FDA due to possible adverse effects, their efficacy and favorable outcomes against cancer have gained attention from scientists around the world who are extensively working to diminish the side-effects and treat cancer effectively.

## 11. Conclusions and Future Applications of PPAR-Gamma Partial Agonists for Precision Oncology

In particular, witnessing the data together tries to update with the scenario that in spite of extensive formulation of ligands for PPAR-γ, the application is limited and largely abandoned by drug companies due to several adverse effects. In light of the evidence, the partial activation of PPAR-γ stands out as an attractive approach, as the ligands would serve as promising drug leads in inducing anti-tumorigenic, anti-inflammatory and anti-diabetic effects. Through their partial agonist effect and preventive mechanism, the ligands would not only exhibit potent activators for transcription, but also be apt for co-activator recruitment with a better implication of genetic expression profiling. The partial activation approach of PPAR-γ may advocate a strategy to combat the deleterious effect of synthetic agonists, TZDs. Owing to their direct involvement in cancer therapy; the ligands may exhibit desired effects by abrogating the side effects caused by certain other agonists. These challenges clearly lie ahead to decipher how such an approach should be carried out as targets and treatments of patients with existing therapies.

## Figures and Tables

**Table 4:** List of PPAR-γ partial agonists and their significant role in cancer therapeutics.

PPAR-γ Partial Agonist	Type of Compound	Effect in Disease	Cell Line	Type of Cancer	References
Deoxyelephantopin	Natural	Apoptosis and cell cycle arrest (G(2)/M)	HeLa	Cervix	[69]
Halofenate	SPPAR-γM	Anti-proliferative effects	MM96L	Melanoma	[226]
Tetrazanbigen	Sterol isoquinoline derivative	Anti-proliferative effects	HepG2 and A549	Liver and lung	[220]
HydroxyCinnamic Acid Derivatives	p-coumaric acid and ferulic acid	Anti-proliferative effects	K562	Chronic Myeloid Leukemia	[222]
Telmisartan	Angiotensin II (Ang II) receptor blocker	Apoptosis and anti-proliferative effects	Caki-1,T24, LNCaP, PC3, DU-145 and NEC-8	Renal, bladder, prostate and testicular	[203]
		Anti-proliferative effects	A549	Lung	[227]

## Data Availability

Not applicable.
